# Synthesis and Anti-Inflammatory and Analgesic Potentials of Ethyl 2-(2,5-Dioxo-1-Phenylpyrrolidin-3-yl)-2-Methylpropanoate

**DOI:** 10.3390/ph17111522

**Published:** 2024-11-12

**Authors:** Abdul Sadiq, Muhammad Arif Khan, Rehman Zafar, Farhat Ullah, Sajjad Ahmad, Muhammad Ayaz

**Affiliations:** 1 Department of Pharmacy, Faculty of Biological Sciences, University of Malakand, Chakdara 18000, Pakistan; 2 Department of Pharmaceutical Chemistry, Faculty of Pharmaceutical Sciences, Riphah International University, Islamabad 44000, Pakistan; 3 Iqra Institute of Health Sciences, School of Pharmacy, Islamabad 44000, Pakistan

**Keywords:** inflammation, ethyl pivalate, cyclooxygenases, pain, molecular docking

## Abstract

**Background/Objectives**: Inflammation and analgesia are two prominent symptoms and often lead to chronic medical conditions. To control inflammation and analgesia, many marketed drugs are in practice but the majority of them have severe side effects. **Methods**: This study involved the synthesis of a pivalate-based Michael product and evaluated it for in vitro COX-1, COX-2, and 5-LOX inhibitory potentials using specific assays. Molecular docking studies were also assessed. Based on the in vitro results, the compound was also subjected to in vivo anti-inflammatory and antinociceptive studies. **Results**: The pivalate-based Michael product (MAK01) was synthesized by an organocatalytic asymmetric Michael addition of ethyl isobutyrate to *N*-phenylmaleimide with an isolated yield of 96%. The structure of the compound was confirmed through ^1^H and ^13^C NMR analyses. The observed IC_50_ values for COX-1, COX-2, and 5-LOX were 314, 130, and 105 μg/mL, respectively. The molecular docking studies on the synthesized compound showed binding interactions with the minimized pockets of the respective enzymes. In a carrageenan model, a percent reduction in edema when administered at 10 mg/kg (a reduction of 33.3 ± 0.77% at the second hour), 20 mg/kg (a reduction of 34.7 ± 0.74% at the second hour), and 30 mg/kg (a reduction of 40.58% ± 0.84% after the fifth hour) was observed. The compound showed a significant response at concentrations of 50, 100, and 150 mg/kg with latency times of 10.32 ± 0.82, 12.16 ± 0.51, and 12.93 ± 0.45 s, respectively. **Conclusion**: In this study, we synthesized a pivalate-based Michael product for the first time. Moreover, based on its rationality and potency, it was found to be an effective future medicine for the management of analgesia and inflammation.

## 1. Introduction

Inflammation serves as a natural defense mechanism of the body against harmful stimuli such as toxins, pathogens, and other damaging factors [1]. Inflammation can be categorized as either acute or chronic [2]. Acute inflammation is typically triggered by tissue damage resulting from injury, harmful substances, or bacterial invasion. It begins quickly and intensifies swiftly, with symptoms potentially lasting for a few days, as seen in cases like cellulitis or acute pneumonia [3,4]. The phase between acute and chronic inflammation, known as subacute inflammation, can persist anywhere from two to six weeks [5], while chronic inflammation, also known as long-term or slow inflammation, persists for extended durations ranging from several months to years [6,7]. According to the World Health Organization (WHO), chronic ailments pose the most substantial risk to human health. In the United States, the prevalence of diseases tied to chronic inflammation is projected to persistently rise for the next three decades [8]. A recent study in 2024 conducted by Mainous indicated that 34.6% of U.S. adults have systemic inflammation. In the year 2014, the Rand Corporation’s more recent findings revealed that nearly 60% of the U.S. population is living with at least one chronic condition. They also found that 42% of Americans have more than one chronic ailment, and 12% of adults are afflicted with five or more such conditions. Globally, the primary cause of three out of five deaths is chronic inflammatory diseases [9]. Chronic conditions involving inflammation not only increase mortality rates, but they also significantly impact quality of life [7,8,10,11]. Persistent or chronic inflammation can lead to tissue damage and health problems, such as in autoimmune diseases, metabolic diseases, or long-term infections, resulting in mortalities worldwide [12,13]. On the other hand, pain is the most frequently reported health problem in both clinical settings and among the general population [14]. Despite efforts to control it, inadequate pain management remains a major and unaddressed issue globally. Acute pain is caused by a specific illness or injury, while chronic pain is considered a disease in its own right [15]. Nociceptive, inflammatory, and neuropathic syndromes are the most common types of acute and chronic pain based on the causes and symptoms [16,17]. Nociceptive pain typically arises from the activation of healthy nerve endings by external triggers or through the discharge of pain-inducing substances. In contrast, inflammatory pain stems from tissue damage and the subsequent activation of inflammation mediators, which amplify the sensation of pain [18,19]. The complex inflammatory cascade may present a potential target for mitigating pain by disrupting the inflammatory process [20]. In cases where acute pain transitions into chronic pain through a process known as central sensitization, both inflammation and the associated pain may persist for extended periods, leading to a chronic pain condition [16,21]. In 2016, it was estimated that around 50 million adults in the United States endured chronic pain. This condition not only had substantial impacts on the health care system but also led to a significant productivity loss [22]. In the year 2021, approximately 20.9% of adult Americans, which corresponds to 51.6 million people, were found to suffer from chronic pain. Moreover, 6.9% of this demographic, representing 17.1 million individuals, experienced what is referred to as high-impact chronic pain. This term denotes chronic pain that drastically inhibits daily activities. Higher rates of chronic pain prevalence were identified among non-Hispanic American Indian or Alaskan Native adults, adults who identify as bisexual, and adults who are divorced or separated [22]. Pain in the back, hip, knee, or foot were among the most highly reported pain conditions [23]. Individuals commonly resorted to physical therapy and massage to alleviate the persistent pain associated with these conditions [24]. People suffering from chronic pain reported impairments in daily functions, including social activities and basic daily tasks [24]. A study across European countries reported a high prevalence of back/neck pain, with 40% of survey participants reporting this pain. It was followed by hand/arm pain, reported by 22% of participants, and foot/leg pain, reported by 21% [25]. Multiple national and international scientific societies have published comprehensive guidelines on the use of various treatments for managing pain. The World Health Organization (WHO)’s “pain ladder” for analgesics has long served as a guide for clinicians in managing pain [26]. The International Association for the Study of Pain (IASP)’s classification of analgesics, which is based on how drugs modify the underlying pathophysiologic mechanisms of pain, may be the most appropriate framework for managing both acute and chronic pain conditions. Despite the complexity and multi-faceted nature of the mechanisms underlying inflammatory pain, which involve both peripheral and central processes, a comprehensive understanding of these mechanisms has yet to be fully established [27,28].

Nonsteroidal Anti-Inflammatory Drugs (NSAIDs) play a significant role in the management of inflammation and pain. However, recent findings on paracetamol have raised questions about its appropriateness in clinical practice, thereby necessitating a re-evaluation of existing recommendations in the clinical practice guidelines [29,30,31]. NSAIDs can be chemically categorized into groups such as phenylpyrazolones, aryl ketones, and selective cyclooxygenase-2 (COX-2) inhibitors, or by their selectivity in inhibiting cyclooxygenase (COX) enzyme isoforms [32,33]. The NSAID diclofenac inhibits both COX enzyme isoforms, while newer drugs show more selectivity towards COX-2, suppressing the generation of prostaglandins (PGs) and other inflammatory mediators [34,35]. This initial response is typically self-limiting and is resolved with the elimination of the inciting stimulus; however, in some cases, the inflammatory process becomes chronic, involving a broader range of mediators that contribute to the development of a range of chronic diseases, such as cancer, atherosclerosis, and rheumatoid arthritis [12,36,37]. Inflammation management involves various treatment options such as NSAIDs, glucocorticoids, slow-acting disease-modifying anti-rheumatic drugs (DMARDs), immunosuppressants, and biologics [38,39]. However, the complexities of the underlying mechanisms and the variability of the diseases make it imperative that the choice of therapeutic options be based on a critical evaluation of the individual patient’s characteristics and the properties of the drugs being considered [40].

NSAIDs have shown adverse effects such as gastrointestinal (GI) toxicity, cardiovascular toxicity, liver injury, and kidney damage [41,42], limiting their full use. Past research has indicated that inhibiting the COX-2 enzyme can effectively manage inflammation, while the inhibition of the COX-1 enzyme may lead to kidney and gastric damage. Traditional NSAIDs inhibit both COX-1 and COX-2 enzymes, often resulting in gastrointestinal upset, local irritation, and gastrointestinal ulcers [43]. Because of these shortcomings, there has been a shift towards developing NSAIDs that selectively inhibit COX-2. However, this has not been without its challenges, as many COX-2-specific drugs have been withdrawn from the market in recent years due to an association with high blood pressure, thrombosis, and heart attacks, especially in COX-2 inhibitors that impede prostacyclin [44,45]. Furthermore, these COX-2-selective inhibitors do not eliminate enteropathy. Numerous studies have demonstrated the vital role of prostaglandins (PGs) related to COX-2 in gastrointestinal protection and the repair of mucosal damage [46]. These findings highlight the complexity and challenges of developing safer and more effective NSAIDs for the management of inflammation and pain. Furthermore, evidence suggests that the COX-1 isoform also contributes to inflammation, challenging the previous hypothesis of the distinct roles of COX-1 and COX-2 isoforms [47,48]. To address the toxicities of NSAIDs, different approaches have been proposed, including prodrug formulations to mask the carboxylic acid functional group, topical drug delivery systems, and combining NSAIDs with anticholinergics, amino acids, carbohydrates, and antioxidants. While non-selective COX inhibitors may have lower toxicity in the cardiovascular system compared to selective COX-2 inhibitors, they have significantly higher toxicity in the GI tract. The development and utilization of NSAIDs must consider their trade-offs and the need for balancing efficacy and safety. Therefore, the main focus for many researchers now is to create a safer NSAID treatment that still provides effective results.

Succinimides, well-known pharmacological pharmacophores, are used in experimental pharmacology and in clinical medicine. These types of compounds are marketed specifically for their anti-convulsant potency [49]. However, the repurposing of drugs is among the major strategies of medicinal chemists. The succinimide derivatives have been previously synthesized for various chemical and pharmacological purposes [50,51]. Due to their diverse structural features, different succinimide derivatives have shown potential effectiveness in inflammation [52], Alzheimer’s [53], hypertension [54], oxidative stress, and diabetes [55,56]. The succinimide can be derivatized at both the C- and N- positions for structural diversity [47,50,54]. The pivalate moiety has been previously reported with potential effectiveness [57,58]. To date, there is no report available on the synthesis of succinimides with the pivalate moiety. Therefore, based on the literature survey and bridging the gaps, we have designed a new derivative of succinimide with the pivalate moiety for potential effectiveness in treating analgesia and inflammation.

## 2. Results

**Scheme 1:**
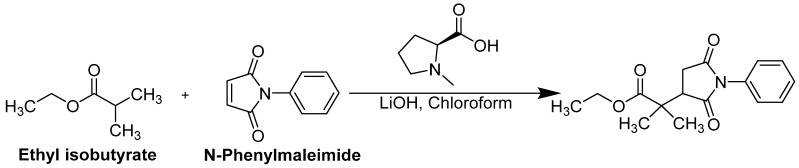
Chemical synthesis of ethyl-2-(2,5-dioxo-1-phenylpyrrolidin-3-yl)-2-methylpropanoate MAK01.

The succinimide product ethyl-2-(2,5-dioxo-1-phenylpyrrolidin-3-yl)-2-methylpropanoate MAK01 was synthesized with an effective synthetic method by a single-step reaction of ethyl isobutyrate with N-phenylmaleimide (Scheme 1). In the presence of methylated L-proline as an organocatalyst, the compound was obtained in a 96% isolated yield in a shorter reaction time of 6 h. Physically, the compound MAK01 appeared as white powder. The compound was soluble in polar organic solvents.

**Figure 1:**
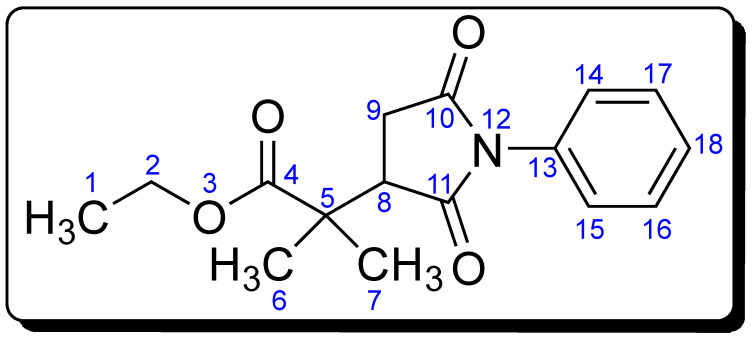
Chemical structure of the compound MAK01.

The compound MAK01 (Figure 1) was characterized and confirmed through ^1^H and ^13^C NMR analyses. The ^1^H NMR, ^13^C NMR, MS, and HPLC spectra are shown in Figures S1 and S2 in the Supporting Information, respectively. The details regarding the assignments of peaks in both ^1^H and ^13^C NMRs are provided in the Supporting Information (Table S1). A monosubstituted benzene ring normally gives three different splitting patterns. The protons at position 16 and 17 appeared as multiplets in the most downfield area in the chemical shift range of 7.44 to 7.49 ppm. Similarly, a multiplet at 7.37–7.42 ppm was observed for the para-aromatic proton at position 18. The ortho-substituted aromatic protons appeared in the form of multiplets in the range of 7.28 to 7.34 ppm. The proton at position 8 gave a doublet of doublet at 4.40 ppm having coupling constant values of 5.27 and 9.52 Hz. Similarly, a quartet (J = 7.14) was observed for the proton at position 2 at a chemical shift of 4.24 ppm. The two diastereotopic protons at position 9 gave two distinct doublets of doublets with a large coupling constant of 18.42 Hz due to geminal splitting. These two doublets of doublets were observed at chemical shift values of 3.02 and 2.80 ppm, respectively. Two distinct singlets were observed for methyl groups at position 6 and 7 at chemical shift values of 1.29 and 1.23 ppm, respectively. The methyl group at position 1 gave a triplet (J = 7.51 Hz) due to the presence of two neighboring Hs at position 2. This triplet was observed in the upfield area of 1.18 ppm. The values calculated for C_16_H_19_NO_4_ were as follows: C, 66.42; H, 6.62; N, 4.84; and O, 22.12. The observed values were as follows: C, 66.54; H, 6.60; and N, 4.81.

**Figure 2:**
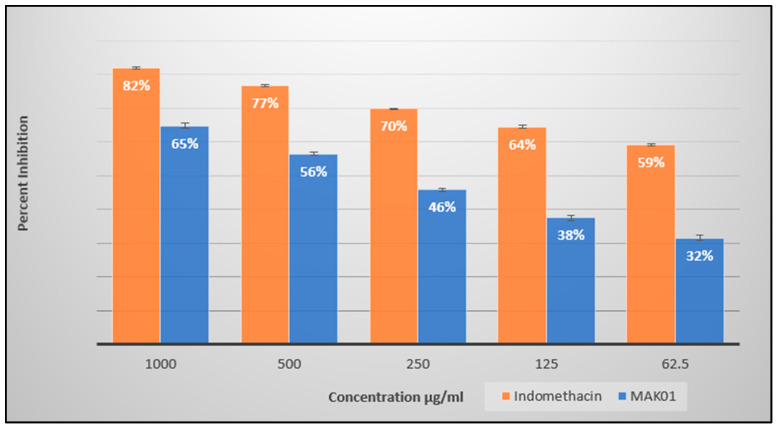
The percentage inhibition of COX-1 activity by various concentrations of the compound MAK01; data points represent the mean inhibition observed at each concentration, highlighting the compound’s potential as a COX-1 inhibitor.

The evaluation of the anti-cyclooxygenase-1 (COX-1) activity of the newly synthesized pivalate-based Michael product was performed, and it was compared with the anti-inflammatory drug, indomethacin. COX-1 is a critical enzyme in the inflammatory process, which converts arachidonic acid into a variety of inflammatory mediators, including interleukins, PGs, leukotrienes, and cytokines. These mediators lead to the development of symptoms such as pain, edema, and redness. The in vitro results indicated that the newly synthesized compound MAK01 had significant anti-COX-1 activity. The compound showed high inhibition levels of 64.79%, 56.45%, 45.75%, 37.51%, and 31.53% at concentrations of 1000 to 62.50 μg/mL, with an IC_50_ of 314 μg/mL. These results demonstrate that MAK01 exhibits moderate anti-COX-1 activity, suggesting a potential anti-inflammatory effect. The inhibition of COX-1 by MAK01 was compared to the inhibition by the reference drug, indomethacin, shown in Figure 2.

**Figure 3:**
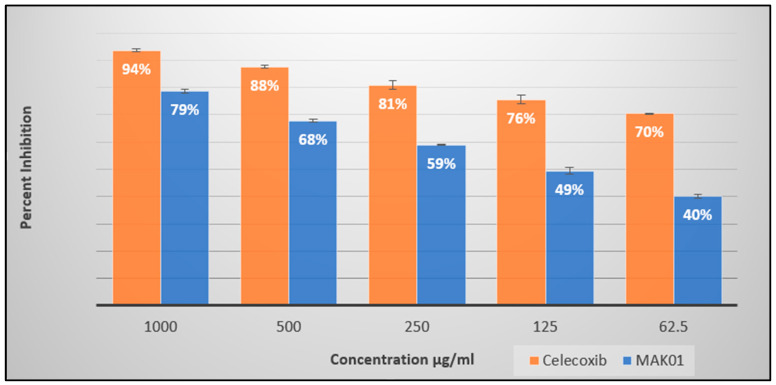
The percentage inhibition of COX-2 activity at different concentrations of the compound MAK01, showing a dose-dependent inhibition of COX-2 enzymes.

The synthesized compound (MAK01) was evaluated for its potential to inhibit the COX-2 enzyme, involved in the progression of inflammatory response. An important function of COX-2 is the transformation of arachidonic acid into several inflammatory mediators, including prostaglandins, leukotrienes, interleukins, and other cytokines. These mediators are responsible for causing symptoms of inflammation such as pain, swelling, redness, and other related symptoms. The in vitro study compared the efficacy of MAK01 with celecoxib in inhibiting COX-2 activity. The results were promising as MAK01 showed a significant inhibition of COX-2 at concentrations of 1000 and 500 μg/mL, as displayed in Figure B. The inhibitory potential of MAK01 was found to be potent, with an IC_50_ of 130 μg/mL, and inhibitions of 78.70, 67.80, 58.90, 49.40, and 40.10% at different concentrations, as shown in Figure 3.

**Figure 4:**
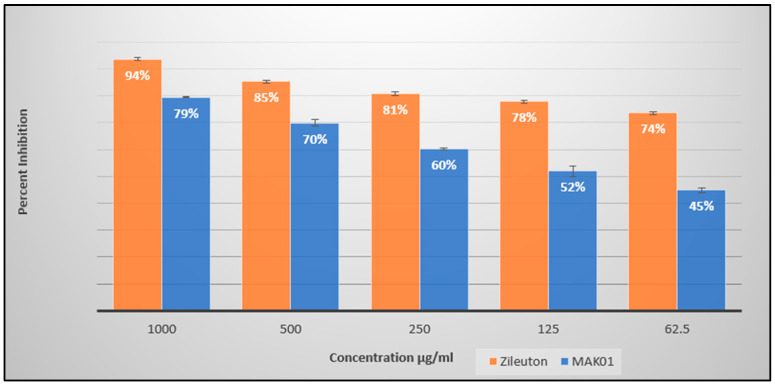
The percentage inhibition of 5-LOX by the compound MAK01 in vitro is presented here; each data point reflects the inhibition level achieved with increasing concentrations of MAK01, indicating its potential as a 5-LOX inhibitor.

These results indicate that MAK01 has the potential to be a selective anti-inflammatory agent as it exhibits a greater inhibitory effect on COX-2 compared to COX-1. The 5-lipoxygenase (5-LOX) enzyme plays a key role in the production of inflammatory leukotrienes and other cytokines through the metabolism of arachidonic acid. In the laboratory assay, the new pivalate-based Michael compound was assessed for its ability to inhibit 5-LOX, and the results were significant. The compound (MAK01), used at higher concentrations, like 1000 and 500 μg/mL, considerably inhibited the 5-LOX enzyme. The suppression of the enzyme was significant at these doses. This suggests that the compound may possess potential anti-inflammatory activity. The synthesized compound demonstrated inhibitory potency at various concentrations. It showed inhibitions of 79.40, 69.90, 60.20, 51.90, and 44.80% at concentrations of 1000, 500, 250, 125, and 62.50 μg/mL, respectively. Using these inhibitory percentages, the calculated IC_50_ value was 105 μg/mL. These results highlight the potential of this compound in inhibiting the 5-LOX enzyme at varying concentrations. Inhibiting the 5-LOX enzyme is a key treatment strategy for many NSAIDs, including zileuton. This highlights the significance of 5-LOX inhibition in the realm of anti-inflammatory drug development. Finally, the results of the 5-LOX inhibition assay were compared with the inhibitory potential of zileuton, a recognized anti-inflammatory drug. This comparison is illustrated in Figure 4 and offers valuable insights into the efficacy of the synthesized compound.

**Figure 5:**
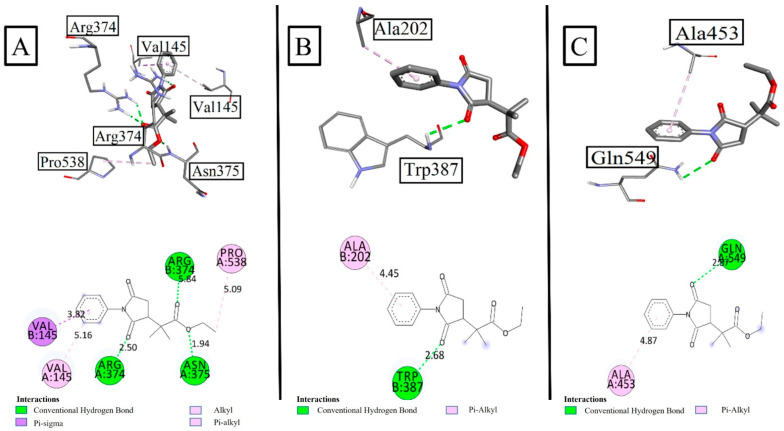
(**A**–**C**)**:** Interaction of synthesized compound (MAK01) with COX-1, COX-2, and 5-LOX.

To assess the pharmacological behavior of the synthesized compound after interacting with targeted proteins, the docking studies were performed to understand the various interaction parameters between the enzyme and the ligand. The findings demonstrated that the compound (MAK01) exhibited significant binding energy. Specifically, it presented a value of −7.7 Kcal/mol when interacting with COX-1, −7.2 Kcal/mol when interacting with COX-2, and −7.1 Kcal/mol when interacting with 5-LOX. The interaction of the synthesized compound with COX-1, COX-2, and 5-LOX was further analyzed and is shown in Figure 5A–C.

**Figure 6:**
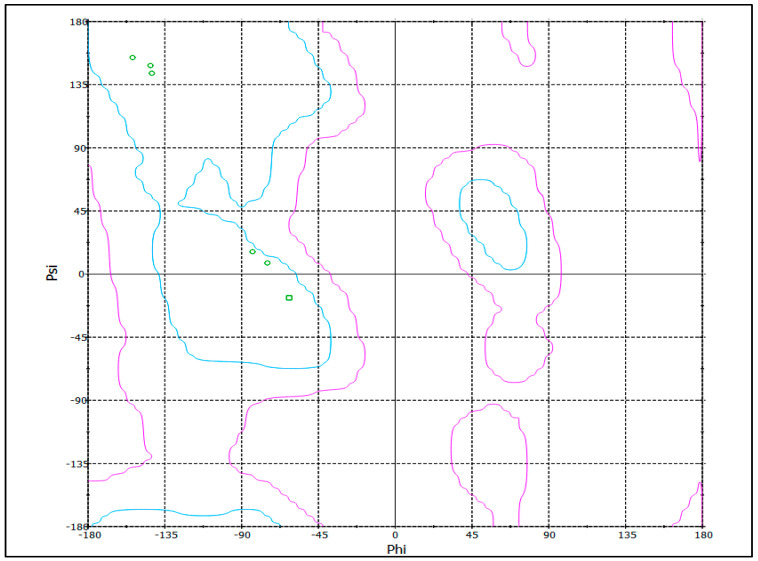
Ramachandran plot showing the behavior of the synthesized compound (MAK01) inside the active site of cyclooxygenase-I. Pink lines indicating the Allowed region, while blue lines indicate the appearance of **MAK01** in allowed regions.

These results offer an in-depth look into the pharmacological interactions of the compounds, providing valuable information for future development and optimization. The performance of the synthesized chemical moiety is demonstrated in a Ramachandran plot, as shown in Figure 6. Table 1 elaborates the binding interaction of the pivalate-based Michael product with the targeted proteins, along with reference drugs.

The physiochemical properties of the synthesized compound gave a detailed picture of ligand behavior inside the body. The compound showed good oral bioavailability and has the capability to cross the blood–brain barrier. Furthermore, the topological polar surface area of this compound was found to be 63.68 A2. The results for the pharmacokinetic behavior were compared with the reference drug indomethacin. All the results are elaborated in Table 2.

In the toxicity study, the compound was evaluated for its impact on test groups of mice. The results showed that there were no adverse effects or mortalities observed at doses lower than 1000 mg per kg body weight. Furthermore, the mice did not show any signs of vision impairment; changes in the heart rate, skin, fur, or breathing; gastrointestinal discomfort; drowsiness; tremors; and more. However, at doses higher than 1000 mg per kg, the mice displayed distress, agitation, convulsions, discomfort, and abnormal behavior. As a result of the study, less than half of the mice were alive after 4 weeks. The results indicated a lethal dose close to 1000 mg per kg of body weight. This estimation is according to the standards outlined by the Organization for Economic Cooperation and Development (OECD). The OECD guidelines suggest a lethal dosage range from 300 to 2000 mg per kg body weight is considered safe for evaluation purposes and falls under category 4 doses, as presented in Table 3.

The anti-inflammatory effect of MAK01 was studied in a carrageenan-induced paw edema animal model. The carrageenan-induced inflammation process was a two-phase event, with the first phase occurring in the first two hours and involving the release of histamine and similar substances, and the second phase taking place two to five hours later and involving the release of leukotrienes, lysosomes, prostaglandins, and proteases. After acclimatization, the mice were administered carrageenan to induce edema and then treated with MAK01 and a control drug in various doses (10, 20, and 30 mg/kg) through oral route administration. The outcomes revealed that MAK01 considerably decreased inflammation induced by carrageenan in the mice’s paws. This was particularly noticeable in the reduction in edema when administered at 10 mg/kg (a reduction of 33.3 ± 0.77% at the second hour), 20 mg/kg (a reduction of 34.7 ± 0.74% at the second hour), and 30 mg/kg (a reduction of 40.58 ± 0.84% after the fifth hour). Aspirin, used as the positive control at 10 mg/kg, demonstrated strong anti-inflammatory activity. It managed to maintain the highest level of inhibition (57.57 ± 1.23%) even beyond the 5 h mark. These results are presented in Table 4. There was no difference in the percent inhibition activity across both male and female mice.

The results of the hot plate test, which measured the antinociceptive potential of the newly synthesized compounds compared to the reference drug morphine, are outlined in Table 5. MAK01 was found to have dose-dependent pain reduction abilities. The response was monitored every 30 min up to 150 min and the compounds showed a significant decrease in pain at different time intervals. The antinociceptive effect of the compound was assessed through measuring the latency time. The latency time was defined as the duration for which a mouse was able to tolerate the heat on a hot plate. The compound MAK01 showed the most significant response at concentrations of 50 mg/kg, 100 mg/kg, and 150 mg/kg, with latency times of 10.32 ± 0.82 s, 12.16 ± 0.51 s, and 12.93 ± 0.45 s, respectively. The results indicate a significant pattern of antinociceptive response for the compound MAK01, as presented in Table 5. The difference in sexes did not affect the outcomes of the hot plate analgesia assay.

## 3. Discussion

Pain and inflammation are two physiological responses that can arise in response to a variety of medical conditions [59]. The process of inflammation within the body can further complicate other comorbidities [60,61]. To combat the inflammatory process, varieties of synthetic and natural substances are in practice [62]. In recent times, there has been an increasing focus on developing new synthetic compounds that have better antinociceptive and anti-inflammatory properties than traditional medicines. In comparison to the natural products, synthetic drugs have two major advantages which have dominated their exploration as drug molecules. The Me Too drug concept for the synthesis of more functional analogs supplements the reliability of repetition with practical yield [63]. Previously, the pivalate pharmacophore was mentioned/reported in only two papers on anti-inflammatory studies [57,58]. Keeping in mind the Me Too drug concept and the literature information on the pivalate pharmacophore, we were also interested in the synthesis of new molecule/s with the pivalate pharmacophore. To achieve interactions in the minimized pocket of COX/LOX enzymes, we set the reaction conditions for the target molecule/s. It was also very practical to achieve a direct C-C bond formation through a Michael addition [64]. Evidently, we obtained MAK01 using a single-step organocatalytic approach with several functionalities which can increase the possibility of binding with the specific protein target. Obviously, as mentioned in our in silico portion, our compound has shown overwhelming outcomes.

Commonly prescribed treatments for pain and inflammation, such as NSAIDs, opioid-based analgesics, and steroidal medications, are recognized in the medical field. However, these medications have a number of known side effects such as stomach complications [65]. The marketed drugs with COX-2 selectivity are preferred for patients having stomach complications [66]. Our synthesized compound MAK01 exhibited an IC_50_ value of 130 μg/mL against the COX-2 enzyme, which is 3-fold more selective compared to COX-1. This shows that our compound will be safe for patients having stomach issues. Furthermore, MAK01 was also found to be very safe when used in experimental animals. Furthermore, in mice, the carrageenan-induced paw edema assay revealed that MAK01 had a significant impact on edema reduction. Interestingly, the edema reduction was similar to the control drug, aspirin, further highlighting the potential therapeutic effects of the compound. These collective findings suggest that these newly synthesized compounds could effectively mitigate pain and inflammation. It also indicates a promising new potential therapeutic agent for other medical conditions caused by inflammation.

## 4. Materials and Methods

### 4.1. Chemicals and Instruments

The chemicals used in this project were chloroform (Reg. No.: 104453) from Merck (Darmstadt, Germany), Dimethylformamide (Reg. No.: 15445) supplied by Sigma-Aldrich (St. Louis, MO, USA), and ethyl isobutyrate (Reg. No.: 373203) from Thermo Fisher Scientific (Waltham, MA USA). Further down in the list were N-substituted maleimide (Batch No.: 829345) provided by Acros Organics, nitro-olefin (Reg. No.: 63245) from Alfa Aesar (Haverhill, MA, USA), and Ethanol (Jefferson, WI, USA) (Batch No.: V002345) supplied by BDH Chemicals (Dubai, United Arab Emirates). Also used were COX-2 enzymes (Batch No.: G5234) from Cayman Chemical (Ann Arbor, MI, USA), an arachidonic acid substrate (Reg. No.: N45354) from Sigma-Aldrich, and 5-LOX enzymes (Batch No.: A3457) supplied by Cayman Chemical. Additionally, the research used Hydrochloric acid (HCl, Reg. No.: 327245) from Merck, Isopropyl alcohol (Reg. No.: 435645) supplied by Honeywell (Charlotte, NC, USA), and glutathione (Reg. No.: 62345) from Sigma-Aldrich. The experiment made use of TLC plates (Batch No.: Z122345) from Merck, celecoxib (Lot No.: S8632) supplied by Pfizer (New York, NY, USA), and TMPD (Tetramethyl-p-phenylenediamine dihydrochloride, Batch No.: A82456) from Sigma-Aldrich. Lastly, DMSO (Lot No.: D5646) from Sigma-Aldrich, KH_2_PO_4_ (Reg. No.: P34562) supplied by Merck, carrageenan (CAS No.: 9023-73-8) from Sigma-Aldrich, and Dinitrosalicylic acid (Batch No.: 834568) from Sigma-Aldrich were used. Other chemicals consisted of Tween 80 (Reg. No.: P7244) from Sigma-Aldrich, COX-1 enzymes (Batch No.: A4570) supplied by Cayman Chemical, indomethacin (Reg. No.: D45762) from Sigma-Aldrich, and zileuton from Abbot Laboratories (Chicago, IL, USA). The research also applied a linoleic acid substrate (Batch No.: N7347) from Sigma-Aldrich, aspirin (Lot No.: 72343) supplied by Bayer (Leverkusen, Germany), and a solution of normal saline from Otsuka Pakistan Ltd. (Karachi, Pakistan).

Various instrumental analyses were also used in this study. A UV spectrophotometer (Model UV-1800) manufactured by Shimadzu, Kyoto, Japan, was used in the study. Additionally, a German-made rotary evaporator with digital functions (Model Hei-VAP) from Heidolph Instruments (Schwabach, Germany) was utilized. The study also employed an incubator manufactured by Memmert, Schwabach, Germany. Several other instruments were utilized in the research process. These included a Korean Daihan scientific UV lamp, type ST001045, and a German-made Type ABS220-4 Electronic Balance. Type EOL ECX 400 Nuclear Magnetic Resonance (NMR) equipment was used, along with a hot plate and column chromatography equipment. Disposable syringes were employed in the study. These were sourced from Shifa (Islamabad, Pakistan), with a Lot Number of SSYJXSCX3421/0543. The use of diverse chemicals and an array of equipment ensured the success of the study.

### 4.2. Synthesis of Pivalate-Based Michael Product

The synthesis of the pivalate-based Michael product, ethyl 2-(2,5-dioxo-1-phenylpyrrolidin-3-yl)-2-methylpropanoate (MAK01), began with the combination of 2.0 equivalent (2.0 mmol, 268.6 μL) of ethyl isobutyrate, a catalytic quantity of N-methyl-L-proline (12.9 mg, 10 mol%), and lithium hydroxide (3.4 mg, 10 mol%) in 1.0 mL of chloroform (1M), followed by the incorporation of 1.0 equivalent of *N*-phenylmaleimide (1.0 mmol, 173.2 mg); the mixture was then subjected to consistent agitation for proper integration. To monitor the progress of the reaction, TLC analysis was employed. At completion of reaction, it was diluted with distilled water. Next, the organic layer was extracted three times, each time using 5 mL portions of chloroform. This resulted in multiple extracted organic layers that were then consolidated. They were then dried with anhydrous sodium sulfate and filtered. Finally, the purification process was started by using column chromatography. The solvents used in the column chromatography were n-hexane and ethyl acetate. The process was initiated with non-polar n-hexane, and we gradually increased the polarity by adding ethyl acetate at every 100 mL interval until the pure compound was eluted [64].

### 4.3. In Vitro Studies

#### 4.3.1. Cyclooxygenase-1 (COX-1) Inhibitory Potential

The inhibitory potential of synthesized MAK01 was evaluated using a COX-1 inhibitory assay [47]. This utilized a solution of COX-1 enzymes derived from sheep, adhering to previous protocols. The COX-1 enzyme solution was prepared with a specific concentration. The concentration was set at 0.7–0.8 μg per 10 μL. The activation of the enzymes was achieved by taking and mixing 10 μL and 50 μL of the COX-2 enzyme solution and a co-factor solution, respectively. This mixture was then cooled at 4 °C in an ice bucket for a duration of five to six minutes. As for the preparation of the co-factor solution, it began with 0.1 M Tris buffer, which was maintained at a pH of 8.0. To this buffer, 1 mM of hematin was added. Next, 0.9 mM of glutathione was mixed into the solution. Further, the solution was enriched with 0.24 mM of TMPD. After the co-factor solution, specific test samples were prepared. These samples were of a certain concentration and diluted to 20 μL in volume. These test samples were then mixed with 60 μL of the COX-2 enzyme solution. Once the mixture was prepared, it was left undisturbed at room temperature. The duration for this standing period ranged from five to ten minutes. Subsequent to this resting period, 20 μL of 30 mM arachidonic acid was added. This addition served to initiate the reaction within the solution. Post initiation of the reaction, the solution underwent an incubation period. The solution was kept at a temperature of 37 °C. The incubation was allowed to continue for a total of 15 min. The final solution underwent an absorbance measurement. This was performed using a UV–visible spectrophotometer. Specifically, the measurement was made at a 570 nm wavelength. Similarly, the test samples in various concentrations were processed and analyzed using the same procedure. Using the absorbance value over time, the inhibition of the COX-1 enzyme was quantified.

In this experiment, indomethacin was used as a comparative option because it has greater selectivity for the inhibition of the cyclooxygenase-1 enzyme and is commonly used in a variety of inflammatory conditions, including arthritis. Using the following formulation, the percent inhibition for each sample was calculated.

$$\%\;Inhibition=\frac{Abs.\;test\;sample-Abs.\;background}{Abs.\;solvent\;blank-Abs.\;background}\times 100$$

#### 4.3.2. Cyclooxygenase-2 (COX-2) Inhibitory Potential

The inhibitory potential of synthesized MAK01 was assessed using the COX-2 inhibitory protocol [47]. The process started with the preparation of a COX-2 enzyme solution with a concentration of 300 U per ml. The activation of the enzymes was achieved by taking and mixing 10 μL and 50 μL of the COX-2 enzyme solution and a co-factor solution, respectively. Once mixed, this solution needed to be chilled. The chilling process was carried out at 4 °C in an ice bath. The duration for this cooling phase was set at five to six minutes. The co-factor solution used in the process had a specific composition. It was made up of 0.1 M Tris buffer with a pH of 8.0, 1 mM of hematin, 0.9 mM of glutathione, and 0.24 mM of TMPD in a phosphate buffer. Following the initial steps, a test sample was prepared. The volume of this sample was fixed at 20 μL. The 20 μL sample was mixed with 60 μL COX-2 solution. After combining them, the sample and enzyme solution were kept at ambient temperature. The solution was allowed to rest for a duration from five to ten minutes. To start the reaction, 20 μL of 30 mM arachidonic acid was incorporated. Upon adding the acid, the solution was subjected to an incubation period. This period lasted for 15 min at a temperature of 37 °C. Post incubation, HCl was added to stop the reaction. The absorbance of this final solution was then measured. This was carried out using a UV–visible spectrophotometer at a specific 570 nm wavelength. Similarly, the above procedure was used for all the other concentrations of the test sample. The COX-2 enzyme inhibition was calculated as per the below equation. For comparative assessment, the NSAID celecoxib (often used for arthritis as an anti-inflammatory drug) was utilized in the experiment. Using the below formula, the percent inhibition of each sample was calculated.

$$\%\;Inhibition=\frac{Abs.\;test\;sample-Abs.\;background}{Abs.\;solvent\;blank-Abs.\;background}\times 100$$

#### 4.3.3. Evaluation of Selectivity Index

Using established protocols, the inhibitory potency of MAK01 against both COX-1 and COX-2 enzymes was assessed. This evaluation allowed for the determination of the selectivity of these new compounds. The IC_50_ values for both COX-1 and COX-2 were then used to calculate the selectivity index of the compound.

#### 4.3.4. 5-Lipooxygenase (5-LOX) Inhibitory Assay

The activity of MAK01 against the 5-LOX enzyme was investigated using a human recombinant form of 5-LOX [47]. To begin the 5-LOX activity assay, a 10,000 U/mL solution of 5-LOX was prepared in a phosphate buffer with a pH of 6.3. A range of concentrations of the test compounds in the same buffer were prepared for assay. As a substrate for 5-LOX, linoleic acid (80 mM) was diluted with ethyl alcohol and used in the assay. The experiment involved mixing the same amount of both the enzyme solution and the test sample, such as mixing 0.25 mL 5-LOX with 0.25 mL of the compound, both dissolved in buffer. The mixing was followed by incubation at 25 °C for 5 to 10 min. Subsequently, 0.10 mL of the linoleic acid solution was added and allowed to incubate for another 5 min. The absorbance was measured using a UV–visible spectrophotometer at a wavelength of 234 nm, and the percent inhibition was calculated based on the absorbance readings. The same procedure was repeated for different concentrations of the test samples. Zileuton, as a reference drug, was used in comparisons. Using the following formula, the percent inhibition for each sample was calculated:

$$\%\;Inhibition=\frac{Abs.\;of\;Control-Abs.\;of\;Sample}{Abs.\;of\;Control}\times 100$$

### 4.4. Molecular Docking Study

An examination of the binding properties of the synthesized compound with chosen macromolecules was conducted through a computational study. This study was carried out using PyRx software as an integral part of our method. This software was enhanced by integrating it with Auto Dock Vina 1.2.2, which broadened our investigative scope. The three-dimensional structures of the synthesized compounds we investigated were initially drafted. To achieve this, we utilized the latest version of ChemDraw 20.0, renowned for its accuracy and efficiency. After being drawn, these structures were saved in the Mol.file format. The structures then underwent modification, with polar hydrogens added to them, and were saved in the pdb format using Discovery Studio Visualizer. The proteins of interest in our study were COX-1, COX-II, and 5-lipoxygenase, which are identified by their PDB identification (ID) numbers 4O1Z, 5F1A, and 3V92. The three-dimensional structures of the proteins were sourced from the widely used RCSB protein data bank, accessible via http://www.rcsb.org (accessed on 31 August 2023) [47]. The protein structures were saved in the pdb format for further use. Their optimization was then achieved by adding polar hydrogens and removing co-crystallized ligands, a process carried out with Bio Discovery Studio Visualizer. After these adjustments, the structures were saved again in the pdb format. The ligands and the optimized protein structures underwent a new process. They were introduced into the PyRx environment for further analysis. Within PyRx, they were converted into their corresponding ligand and macromolecule formats, known as Pdbqt. Subsequently, the grid box was set up; it was configured with a center having the coordinates X: 10.132, Y: 66.509, and Z: 32.9381. The dimensions of the grid box were precisely defined as X: 77.0983, Y: 106.8761, and Z: 105.4147, measured in Angstroms, in the case of COX-I and COX-II, while they was set as random in the case of 5-LOX. The ligands were subsequently docked into the active sites of the receptors, which were defined by identifying co-crystallized ligand pockets. To gain a comprehensive understanding of the best interactions, the types of bonds, amino acid residues, and bond distances were examined using various parameters. These details were visualized in different colors through the use of Discovery Studio Visualizer and Pymol 1.8.

### 4.5. Physiochemical Property Prediction and ADMET Properties

The behavior of the synthesized compound MAK01 inside the body, including pharmacokinetic parameters and drug likeliness, Lipinski’s rule of five, was analyzed using the SWISS ADME virtual prediction tool [5,27]. The compound was examined via the Molinspiration online tool. According to Lipinski’s rule of five, a compound should have less than certain amounts of various molecular characteristics to be considered as a good drug candidate. Hydrogen bond donors should be less than or equal to 5, hydrogen bond acceptors should be less than or equal to 10, the molecular weight should be less than or equal to 500 Dalton, and the partition coefficient (ClogP) should be below 5. (Nogara PA, Saraiva RD, Caeran Bueno D, Lissner LJ, Lenz Dalla Corte C, Braga MM, Rosemberg DB, Rocha JB.) Virtual screening of the acetylcholinesterase inhibitors was conducted using Lipinski’s rule of five and the ZINC data bank.

### 4.6. In Vivo Studies on the Pivalate-Based Michael Product

#### 4.6.1. Experimental Animals

In this animal study, male albino mice with an average weight of 27.5 g (25 to 30 g) were employed. The mice were obtained from the National Institute of Health located in Islamabad, Pakistan, and transported to the laboratory under appropriate conditions. The laboratory was meticulously controlled to ensure a consistent environment for the study. The temperature was kept at a steady 25 °C, with an acceptable fluctuation of plus or minus 2 °C. A relative humidity level of 50%, allowing for a variance of 5%, was also maintained, alongside a strict 12 h light/dark cycle. Throughout the experiment, the mice participating in the study were treated with the utmost care, receiving a balanced, standard diet and having continual access to water. All experiments were performed with the utmost care and in accordance with ethical standards, and were approved by the Institutional Research Ethical Committee (IREC) at the University of Malakand, Pakistan (Ref. No.: REC/PHAR-UOM/059A). This specific committee operates under and follows the 2008 by-laws for animal experimentation and follows strict protocols for the humane treatment of animals. To minimize stress and ensure the ethical handling of the mice, each animal was used only once in the study and was carefully euthanized following established procedures [47].

#### 4.6.2. Acute Toxicity

The synthesized compound was assessed for its acute toxicity using mice sourced from the National Institute of Health in Pakistan. The mice for the experiment were categorized into several groups. Some groups were designated to receive the test samples, and another was set as a control group. The test compounds were given to the mice orally, with the dosage varying between 25 mg/kg and 2000 mg/kg of the mouse’s body weight. These compounds were first dissolved in normal saline before being administered. Each dose group consisted of 06 mice, split equally between males and females, with 02 males and 02 females receiving MAK01, and the remaining 02 mice receiving normal saline as the control group. The mice were monitored for a period of 24 h to 4 weeks following the administration of the pivalate-based Michael product. The study closely observed the mice for any irregular behavior, such as ataxia, aggressive reactions, and potential allergic symptoms, among others. Furthermore, any harmful outcomes or lethality were also recorded. These observations and recorded adverse events were compared between the group treated with the test compound and the control group. These comparisons offer valuable insights into the toxicity levels of the newly synthesized compounds [47].

#### 4.6.3. Anti-Inflammatory Potential of the Pivalate-Based Michael Product

The anti-inflammatory activity of MAK01 was evaluated through a carrageenan-induced paw edema model [47]. The mice, with weights ranging from 25 to 30 g, were separated into five distinct groups, these being Vehicle Group 1, Aspirin Group 2, and different concentrations of MAK01 in Groups 3–5. Each of these groups comprised five mice. The negative control group in the experiment received a specific solution comprising normal saline, DMSO, and Tween-80. The mixture contained 94% normal saline, 5% DMSO, and 1% Tween-80, delivered at a concentration of 10 mL per kg. In contrast, the positive control group in the assay received the comparator aspirin at a precise dose of 10 mg/kg. The test samples were administered at doses of 10 mg/kg, 20 mg/kg, and 30 mg/kg, given intra-peritoneally. After 30 min, a 2% carrageenan suspension (0.2 mL) was administered to each mouse in every group via a subcutaneous injection in the hind paw. In the experiment, a plethysmometer was used to precisely measure inflammation and edema. These measurements were taken at multiple time points both prior to and following the carrageenan administration. Specifically, the readings were recorded at 1, 2, 3, 4, and 5 h intervals to monitor the progress of the inflammatory response and paw swelling. These obtained results were then compared against the outcomes from the negative control group. The gathered data from the experiment, relating to the impact of the newly synthesized compounds on paw edema, were carefully processed to derive the percentage of edema inhibition. Using the below equation, the percent inhibition was calculated.

$$\%\;Inhibition=\frac{Ce-Te}{Ce}\times 100$$

In the formula, ‘Ce’ refers to the control group paw edema volume, while ‘Te’ denotes test groups’ paw edema volume.

#### 4.6.4. Antinociceptive Potential of the Pivalate-Based Michael Product

The antinociceptive effect of the synthesized compound (MAK01) was evaluated using a hot plate method [16]. For this purpose, mice were separated into distinct groups. These groups consisted of test and control categories for better evaluation and analysis. The test groups received different doses of the synthesized compounds at concentrations of 50, 100, and 150 mg/kg, administered 30 min prior to the hot plate experiment. The hot plate apparatus was maintained at a temperature of 55 ± 0.2 °C, and each mouse was placed on the plate to measure the response latency, or the time taken for the mouse to react by licking its paw, jumping, or escaping. Careful observations were performed to record the response latency of the mice on the hot plate. These observations took place at multiple time intervals, specifically 30, 60, 90, and 120 min post administration of the synthesized compound. In order to establish a baseline for comparison, a positive control was also included in the experiment. The control received the comparator drug, morphine, administered at a dose of 5 mg/kg. The results of the test groups were analyzed and compared to the positive control to assess the effectiveness of the newly synthesized compound as an analgesic.

### 4.7. Statistical Analysis

The study was conducted following rigorous protocols, conducting each experimental procedure on three separate occasions to ensure the accurate evaluation of results [47]. The collected data were analyzed using descriptive statistics, expressed as the mean ($\bar{X}$) ± the standard error of the mean, SE ($\bar{X}$). Further analysis was performed with SPSS software, utilizing a One-Way ANOVA. To perform multiple comparisons, Duncan’s test was applied. The results were then visualized through graphs generated with GraphPad Prism 9.0 (GraphPad Software©, San Diego, CA, USA).. In order to establish statistical significance, a *p*-value of less than 0.05 was considered.

## 5. Conclusions

In this piece of research, we synthesized a new derivative of succinimide including a pivalate moiety in its structure. The compound was synthesized and developed as a new molecule for the treatment of analgesia and inflammation. The new compound was initially evaluated against the COX-1, COX-2, and 5-LOX enzymes to find out its possible role in blocking the COX/LOX pathways in vitro. Based on the IC_50_ values of the compound MAK01, we further evaluated this compound in in vivo antinociceptive and anti-inflammatory models. The potential outcomes of this study raise the possibility of MAK01 functioning as a multimodal therapeutic agent. This means that the compound possesses both antinociceptive and anti-inflammatory activities that target the inflammatory cascade, as well as the central and peripheral nervous systems. However, further extensive research is necessary to fully understand the exact mechanisms and potential for human applications. The future outlook for this new pivalate-based Michael product includes investigating its pharmacological properties against various medical conditions. This research has shown great potential for the development of new and improved medical treatments for pain and inflammation.

## Figures and Tables

**Table 1:** Binding score of pivalate-based Michael product and comparator drugs with targeted macromolecule.

Compounds	COX-I (4O1Z)	COX-II (5F1A)	5-LOX (3V92)
**MAK01**	−7.7	−7.2	−7.1
Montelukast	--	--	−7.7
Celecoxib	--	−8.3	--
Indomethacin	−7.8	--	--

**Table 2:** Physiochemical properties/ ADME prediction of MAK01.

Physicochemical Properties		
Properties	MAK01	Indomethacin
Molecular weight	289.33 g/mol	357.79 g/mol
No. of heavy atoms	21	25
No. of arom. heavy atoms	6	15
Fraction of Csp3	0.44	0.16
No. of rotatable bonds	5	5
No. of H-bond acceptors	4	4
No. of H-bond donors	0	1
Molar refractivity	81.35	96.14
Topological polar surface area (TPSA)	63.68 A^2^	68.53 A^2^
Lipophilicity		
Consensus log *P*_o/w_	2.17	3.28
Water Solubility		
Log S (ESOL)	−2.69	−4.25
Solubility	5.95 × 10^−1^ mg/mL	2.01 × 10^−2^ mg/mL
Class	Soluble	Moderately soluble
Pharmacokinetics		
GI absorption	High	High
BBB permeation	Yes	Yes
P-gp substrate	No	No
CYP1A2 inhibitor	No	Yes
CYP2C19	Yes	Yes
CYP2C9 inhibitor	No	Yes
CYP2D6 inhibitor	No	No
CYP3A4 inhibitor	No	No
Log *K*_p_ (skin permeation)	−6.74 cm/s	−6.14 cm/s
Drug Likeness		
Lipinski	Yes (0 violations)	Yes (0 violations)
Ghose	Yes	Yes
Veber	Yes	Yes
Egan	Yes	Yes
Muegge	Yes	Yes
Bioavailability score	0.55	0.85

**Table 3:** Acute toxicity evaluation of compound MAK01.

Dose No.	Animals		Doses
	Male	Female	
**I**	3	3	25 mg/kg
**II**	3	3	50 mg/kg
**III**	3	3	100 mg/kg
**IV**	3	3	200 mg/kg
**V**	3	3	300 mg/kg
**VI**	3	3	400 mg/kg
**VII**	3	3	500 mg/kg
**VIII**	3	3	1000 mg/kg
**IX**	3	3	1500 mg/kg
**X**	3	3	2000 mg/kg

**Table 4:** Percent inhibition of paw edema at different intervals.

Administered Sample	Dose	Paw Edema Inhibition in Percent				
		1 h	2 h	3 h	4 h	5 h
Vehicle	10 mL	7.11 ± 0.015	7.63 ± 0.044	7.42 ± 0.032	7.84 ± 0.033	7.80 ± 0.035
Aspirin	10 mg	47.46 ± 0.80	54.42 ± 1.10	55.23 ± 0.73	56.22 ± 1.34	57.57 ± 1.23
**MAK01**	10 mg/kg	30.3 ± 0.77 ^ns^	33.34 ± 0.98 *	19.62 ± 0.64 ***	31.44 ± 0.74 **	31.22 ± 0.84 **
	20 mg/kg	34.7 ± 0.74 ^ns^	34.54 ± 0.76 *	21.42 ± 0.64 **	29.52 ± 0.63 **	31.85 ± 0.32 *
	30 mg/kg	38.74 ± 0.81 ^ns^	39.20 ± 0.86 *	34.4 ± 0.64 **	37.64 ± 0.86 **	40.58 ± 0.84 **

The *p*-values less than 0.05 were considered statistically significant. Values significantly different in comparison to the standard drug: * = *p* < 0.05, ** = *p* < 0.01, and *** = *p* < 0.001. All values are taken as mean ± SEM (n = 3).

**Table 5:** Antinociceptive potential; latency time at different intervals.

Samples	Dose mg/kg	Latency Time (Seconds) on Hot Plate				
		30 min	60 min	90 min	120 min	150 min
Negative Control	---------	03.70 ± 0.42	04.35 ± 0.57	02.93 ± 0.33	02.93 ± 0.50	02.70 ± 0.44
MAK01	50	10.32 ± 0.82 *	9.96 ± 0.16 ***	9.63 ± 0.26 ***	9.49 ± 0.25 **	9.16 ± 0.81 ***
	100	12.16 ± 0.51 ^ns^	11.92 ± 0.71 **	11.76 ± 0.87 ^ns^	11.14 ± 0.91 ^ns^	10.96 ± 0.74 *
	150	12.93 ± 0.45 ^ns^	12.85 ± 0.84 ^ns^	12.42 ± 0.36 ^ns^	11.98 ± 0.74 ^ns^	11.76 ± 0.41 ^ns^
Morphine	5	13.83 ± 0.63	13.52 ± 0.23	13.29 ± 0.78	12.92 ± 0.56	12.59 ± 0.41

The *p*-values less than 0.05 were considered statistically significant. Values significantly different in comparison to the standard drug: * = *p* < 0.05, ** = *p* < 0.01, and *** = *p* < 0.001. All values are taken as mean ± SEM (n = 3).

## Data Availability

Data are available upon request.

## Supplementary Materials

The following supporting information can be downloaded at: https://www.mdpi.com/article/10.3390/ph17111522/s1, Figure S1: 1H NMR spectrum of the compound MAK01. Figure S2: 13C NMR spectrum of the compound MAK01. Figure S3: MS spectrum of the compound MAK01. Figure S4: HPLC chromatogram of the compound MAK01. Table S1: Signal assignment in 1H & 13C NMR.
