# Supplementary material for: Synthesis and Anti-Inflammatory and Analgesic Potentials of Ethyl 2-(2,5-Dioxo-1-Phenylpyrrolidin-3-yl)-2-Methylpropanoate
Source: Pharmaceuticals (Basel). 2024 Nov 12;17(11):1522. doi: 10.3390/ph17111522 (PMC11597207; doi:10.3390/ph17111522)
Supplement: Supplementary file 1 [file pharmaceuticals-17-01522-s001.zip › pharmaceuticals-3205707-supplementary.pdf]

**Table S1:** Signal assignment in $^{1}$H & $^{13}$C NMR.

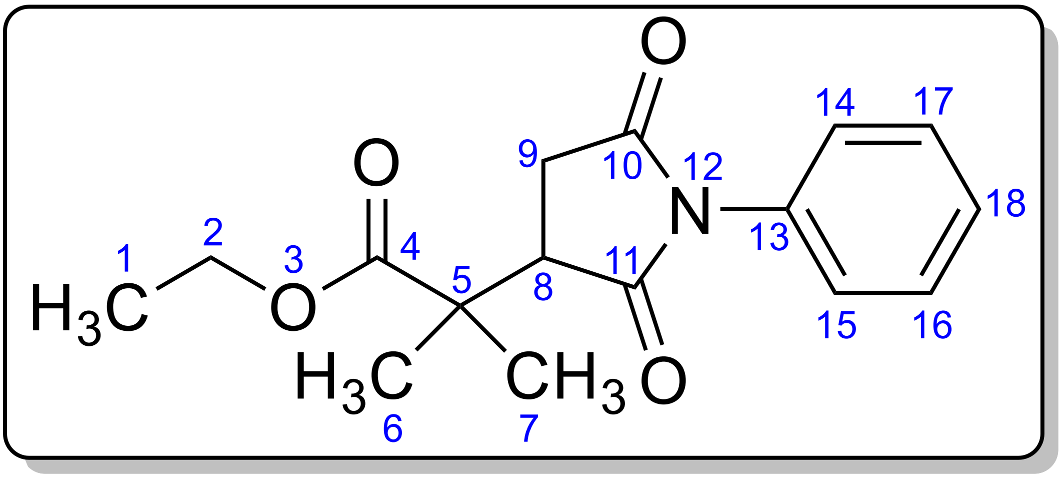

| $^{1}$H NMR | | | | | $^{13}$C NMR | |
|---|---|---|---|---|---|---|
| **S. No** | **Chemical shift** | **Splitting** | **Integration (H)** | **Assignment (H-position)** | **Chemical shift** | **Assignment (C-position)** |
| 1 | 1.18 | t | 3 | H-1 | 16.10 | 1 |
| 2 | 1.23 | s | 3 | H-6 | 17.94 | 6 |
| 3 | 1.29 | s | 3 | H-7 | 18.74 | 7 |
| 4 | 2.79 | dd | 1 | H-9a | 30.09 | 5 |
| 5 | 3.02 | dd | 1 | H-9b | 42.73 | 8 |
| 6 | 4.24 | q | 2 | H-2 | 46.01 | 9 |
| 7 | 4.40 | dd | 1 | H-8 | 62.54 | 2 |
| 8 | 7.29-7.34 | m | 2 | H-16&17 | 126.49 | 16&16 |
| 9 | 7.37-7.42 | m | 1 | H-18 | 128.62 | 18 |
| 10 | 7.44-7.49 | m | 2 | H-14&15 | 129.40 | 14&15 |
| 11 | - | - | - | - | 131.81 | 13 |
| 12 | - | - | - | - | 173.81 | 4 |
| 13 | - | - | - | - | 176.30 | 10 |
| 14 | - | - | - | - | 178.35 | 11 |

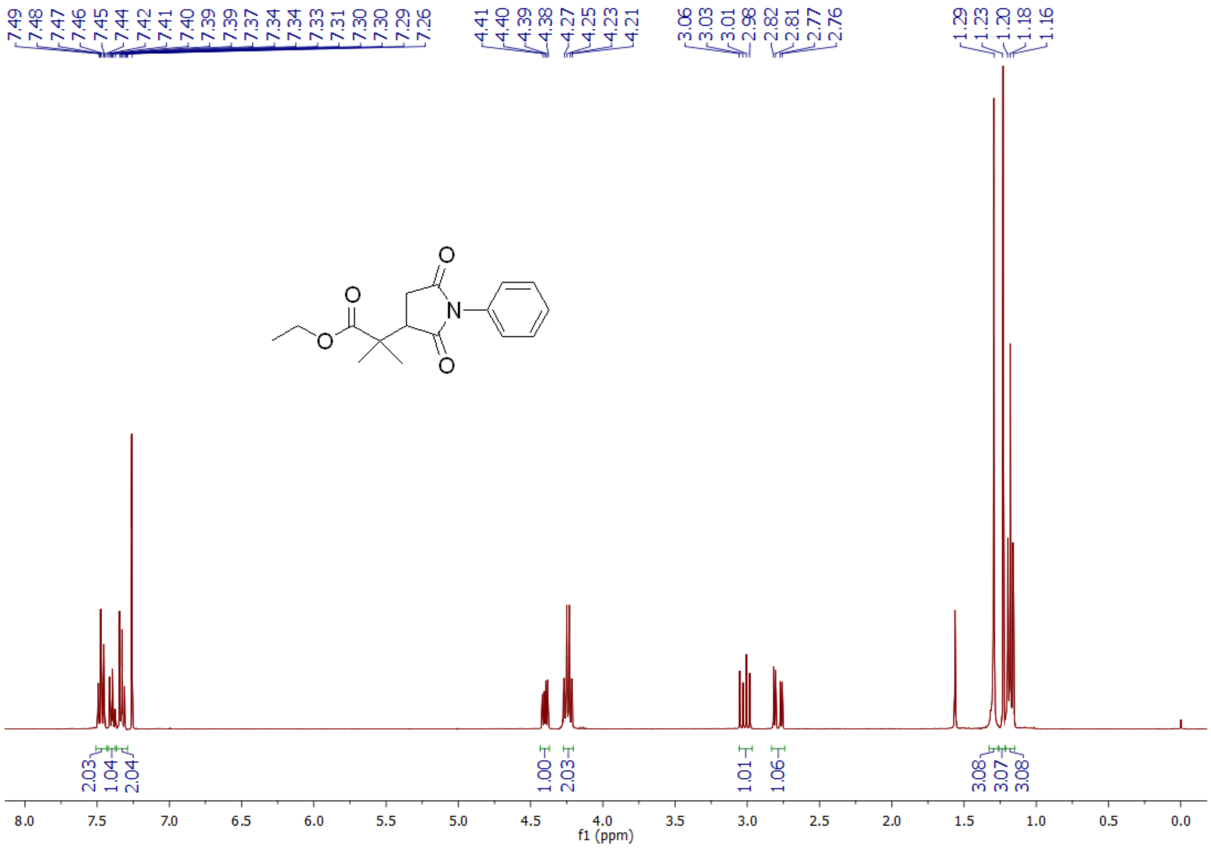


**Figure S1:** $^{1}H$ NMR spectrum of the compound **MAK01**.

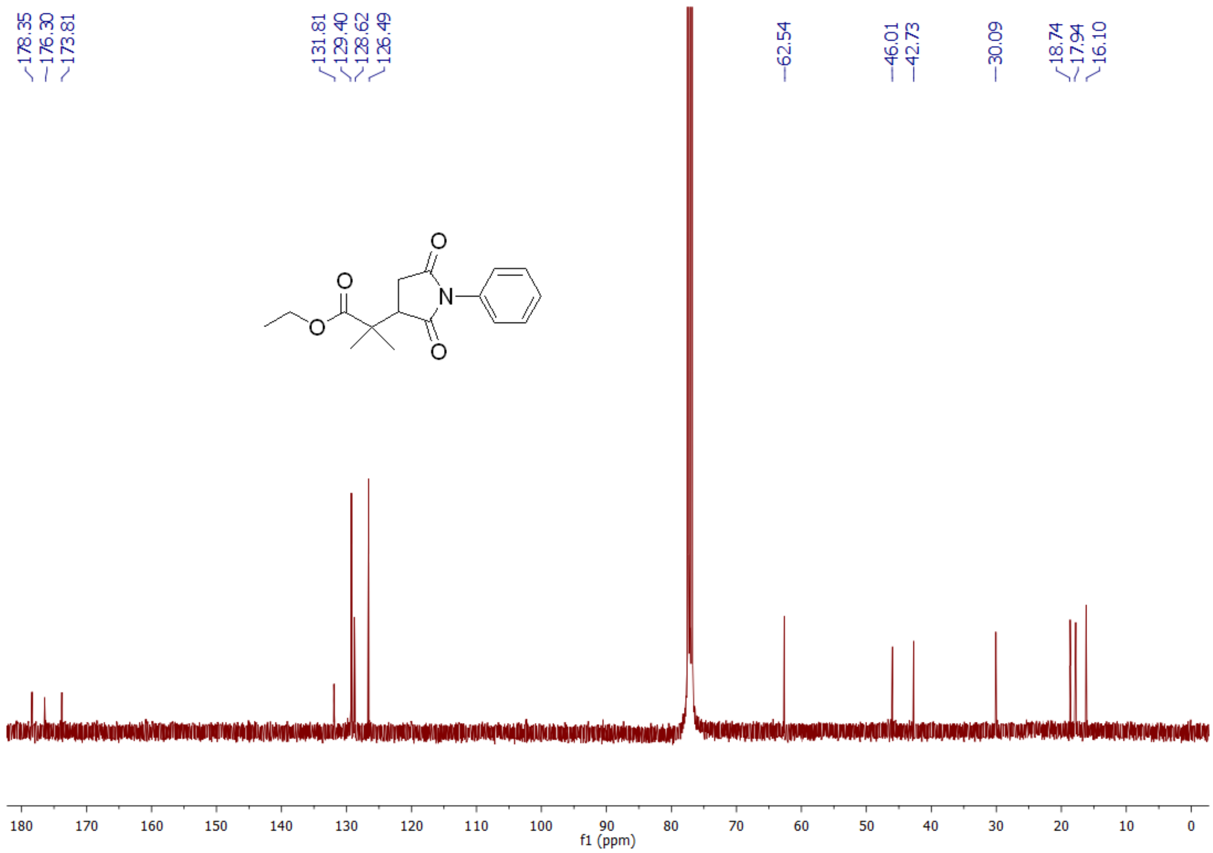


**Figure S2:** $^{13}C$ NMR spectrum of the compound **MAK01**.

# Mass Spectrum SmartFormula Report

**Analysis Info**

| | | | |
|---|---|---|---|
| Analysis Name | F:\Sadiq\25102024\Sadiq Sample 25102024.d | Acquisition Date | 25.10.2024 12:49:37 |
| Method | tune_low-neg1.m | Operator | Bruker |
| Sample Name | Sadiq Sample 25102024 | Instrument | micrOTOF 213750.00129 |
| Comment | Sadiq Sample 25102024 | | |

**Acquisition Parameter**

| | | | | | |
|---|---|---|---|---|---|
| Source Type | ESI | Ion Polarity | Negative | Set Nebulizer | 4.0 Bar |
| Focus | Active | | | Set Dry Heater | 220 °C |
| Scan Begin | 200 m/z | Set Capillary | 4000 V | Set Dry Gas | 4.0 l/min |
| Scan End | 400 m/z | Set End Plate Offset | -500 V | Set Divert Valve | Source |

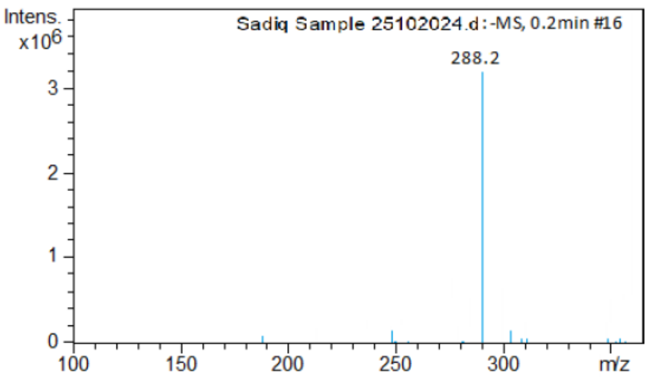


**Figure S3:** MS spectrum of the compound **MAK01**.

**Clarity - Chromatography SW**
DataApex 2006
www.dataapex.com

Chromatogram Info:

| | | | |
|---|---|---|---|
| File Name | : D:\HPLC/MAK01-sample 1 | File Created | : 19/10/2024 12:22:44 |
| PMOrigin | : Acquired | Acquired Date: | 19/10/2024 12:22:44 PM |
| Project | : C:\Clarity Lite\Projects\Work1.PRJ | By | : Dr. Sajjad Ahmad |

Printed Version Info:

| | | | |
|---|---|---|---|
| Printed Version | : 19/10/2024 12:22:44 PM | Printed Date | : 19/10/2024 12:24:47 PM |
| Report Style | : C:\Clarity Lite\Common\Chromatogram.sty | By | : Dr. Sajjad Ahmad |
| Calibration File | : None | | |

Sample Info:

| | | | |
|---|---|---|---|
| Sample ID | : MAK01-sample 1 | Amount | 0 |
| Sample | : 1 | ISTD Amount | : 0.1 |
| Inj. Volume [ml] | : 0 | Dilution | 1 |

| | | | |
|---|---|---|---|
| Method | : MAK01-sample 1 | By | : Dr. Sajjad Ahmad |
| Description | : MAK01-sample 1 | | |
| Created | : 19/10/2024 11:37 AM | Modified | : 19/10/2024 12:22 PM |
| Column | : OD | Detection | : 25nm |
| Mobile Phase | : Heptane : Isopropanol : (90 : 10) | Temperature | : 30 |
| Flow Rate | : 1.0ml/min | Pressure | : 3.6 MPa |
| Note | : | | |

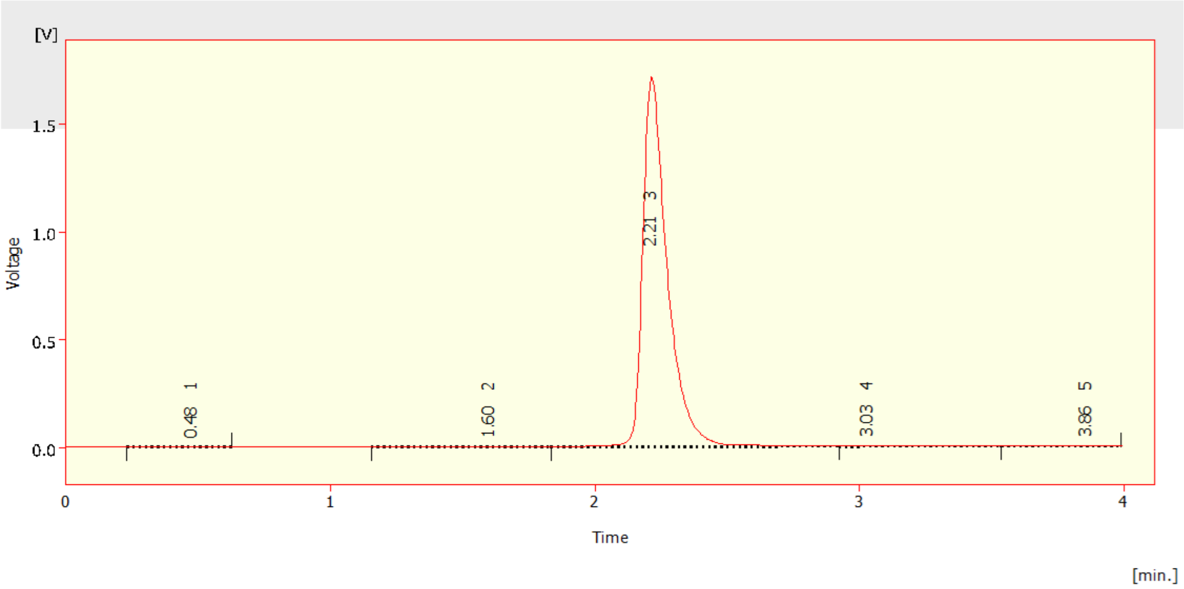


Result Table (Uncal - D:\HPLC/ MAK01-sample 1 - Detector 1)

| | Reten. Time [min] | Area [mV.s] | Height [mV] | Area [%] | Height [%] | W05 [min] |
|---|---|---|---|---|---|---|
| 1 | 0.477 | 6.062 | 0.472 | 0.1 | 0.0 | 0.22 |
| 2 | 1.600 | 27.026 | 1.201 | 0.2 | 0.1 | 0.40 |
| 3 | 2.213 | 11049.851 | 1709.526 | 99.1 | 99.7 | 0.09 |
| 4 | 3.030 | 60.571 | 3.492 | 0.5 | 0.2 | 0.32 |
| 5 | 3.857 | 11.841 | 0.584 | 0.1 | 0.0 | 0.28 |
| | Total | 11155.351 | 1715.274 | 100.0 | 100.0 | |

**Figure S4:** HPLC chromatogram of the compound **MAK01**.
